# Computational sensing of herpes simplex virus using a cost-effective on-chip microscope

**DOI:** 10.1038/s41598-017-05124-3

**Published:** 2017-07-07

**Authors:** Aniruddha Ray, Mustafa Ugur Daloglu, Joslynn Ho, Avee Torres, Euan Mcleod, Aydogan Ozcan

**Affiliations:** 1 Electrical Engineering Department, University of California, Los Angeles, CA 90095 USA; 2 Bioengineering Department, University of California, Los Angeles, CA 90095 USA; 3 California NanoSystems Institute (CNSI), University of California, Los Angeles, CA 90095 USA; 4 Department of Physics, University of California, Los Angeles, CA 90095 USA; 5 College of Optical Sciences, University of Arizona, Tucson, AZ 85721 USA; 6 Department of Surgery, David Geffen School of Medicine, University of California, Los Angeles, CA 90095 USA

## Abstract

Caused by the herpes simplex virus (HSV), herpes is a viral infection that is one of the most widespread diseases worldwide. Here we present a computational sensing technique for specific detection of HSV using both viral immuno-specificity and the physical size range of the viruses. This label-free approach involves a compact and cost-effective holographic on-chip microscope and a surface-functionalized glass substrate prepared to specifically capture the target viruses. To enhance the optical signatures of individual viruses and increase their signal-to-noise ratio, self-assembled polyethylene glycol based nanolenses are rapidly formed around each virus particle captured on the substrate using a portable interface. Holographic shadows of specifically captured viruses that are surrounded by these self-assembled nanolenses are then reconstructed, and the phase image is used for automated quantification of the size of each particle within our large field-of-view, ~30 mm^2^. The combination of viral immuno-specificity due to surface functionalization and the physical size measurements enabled by holographic imaging is used to sensitively detect and enumerate HSV particles using our compact and cost-effective platform. This computational sensing technique can find numerous uses in global health related applications in resource-limited environments.

## Introduction

Herpes is one of the most common viral diseases, which is caused by a DNA based virus, i.e., the herpes simplex virus (HSV). There are two different types of HSV: HSV-1, responsible for oral herpes and HSV-2, which causes genital herpes. It is estimated that >50% of Americans between 14–49 years of age have tested seropositive for this virus^1, 2^. Once contracted, HSV establishes a lifetime latent infection, with periodic outbreaks that lead to painful sores and blisters around the mouth, eye and genitals. Some of the additional complications also include fever and sore throat among others. Recent studies have shed light onto the viral structure of HSV and its interaction with the host cells. The viral DNA is packed inside the capsid, which is surrounded by a layer of proteins called the tegument that is further enveloped in the lipid bilayer. The virus in its dormant state is generally located in the axons of peripheral nervous system neurons and can travel to epithelial cells leading to an outbreak^3–5^. However, the virus can still be transmitted if no active symptoms are present. Additionally it has been well documented that people carrying this virus may not show any symptoms for a long period of time and be unaware of this infection. These factors significantly increase the likelihood of spreading this virus unknowingly. It has been estimated that the total sexually-transmitted-diseases (STD) related medical costs in the US over the last decade was over 15 billion USD, with HSV alone costing more than 500 million USD^6^. This obviously outlines the need for proper and timely screening of HSV to prevent its wide-spread transmission and ensure proper treatment. Patient management studies have clearly shown the benefits of early testing, as it helps in definitive diagnosis, which leads to proper course of therapy as well as counseling for the patients and their significant others^7^.

Currently the two most commonly used clinical HSV diagnostic techniques are viral culture and polymerase chain reaction (PCR). Viral culture involves extraction of the virus from the lesions, using swabs and sterile needles, and culturing them for several days, followed by different assays such as hemagglutination^8^. PCR is an extremely sensitive technique that can be used to detect viruses with great sensitivity and specificity^9^. In addition to PCR, loop mediated isothermal amplification (LAMP) based methods have also been used for the detection of HSV^10^. Serological tests that sense HSV specific antibodies using techniques such as enzyme-linked immunosorbent assays (ELISA)^11, 12^, western blot^13^, antibody-dependent cellular lysis, and radio-immunoassays are also available. Some other HSV detection methods include immunofluorescence microscopy^14^, luciferase immunoprecipitation assay^15^, Tzanck smear^16^ and electron microscopy^8, 17^. Some of these immunoassay based tests, however, can have significant amounts of incorrect results^18, 19^. It was previously reported that the sensitivity and specificity of enzyme based immunoassay techniques compared to culture was 65% and 93%, respectively^20^. Compared to culture, the specificity was close to 100% and the sensitivity varied between 50% and 100% for direct fluorescence immunoassay based approaches^21^. Among the currently used clinical methods, PCR has been shown to have quite high sensitivity and specificity. For example, the commercially available PCR based MultiCode-RTx kit (Luminex, Austin) has a reported sensitivity and specificity of 92.4% and 98.3%, respectively for HSV-1 and 95.2% and 93.6%, respectively for HSV-2^21^. PCR based techniques also have a very good limit of detection, typically ~10 viral particles per assay^22^. However, PCR, along with most of the other techniques, require expert handling and relatively costly and bulky instrumentation, which limit wide-scale availability of these tests, particularly in rural areas and resource limited settings.

Here we present a cost-effective and easy to use mobile platform for HSV-1 detection, using a portable lensfree holographic microscope with pixel super-resolution capabilities. In this label-free sensor design, HSV-1 viral particles, which are ~150–200 nm in size, are detected using a compact holographic microscope after capturing them on specially prepared substrates, using virus-specific antibodies. This approach allows us to utilize antibodies to specifically capture the virus on a substrate and use the computational imaging based microscopic sizing as a secondary method of validation and detection, thus eliminating the necessity for labeling with fluorophores or enzymes. Recently, lensfree holographic imaging has shown promising results for imaging and detection of nanoparticles due to its wide field-of-view (FOV), cost effectiveness and simplicity^23–30^ and in this work we utilize this computational imaging framework for specific and sensitive detection and enumeration of HSV-1 particles. In our holographic microscope, the substrate is illuminated from the bottom using a programmable-array of LEDs and the interference pattern (i.e., the hologram) formed due to the scattered optical wave from the viruses and the non-diffracted wave, is recorded on a CMOS imager chip placed close to the sample, with sub-mm gap between the viral particles and the sensor active area. The optical signal from each virus particle is further boosted by using nanolenses, which are self-assembled around the viruses using polyethylene glycol (PEG) vapor. A unique feature of this lensfree imaging platform is that both amplitude and phase information of the objects are digitally reconstructed from the holographic diffraction patterns captured by the image sensor chip. In this computational microscope, a set of e.g., 20 sub-pixel shifted images is captured and then a super-resolved hologram is obtained by using a lateral-shift based pixel super-resolution method^31–33^. Following the reconstruction of the super-resolved hologram, quantitative phase information of the captured particles is also obtained. Based on the information of this phase channel, the size of the particles can be determined using a calibration curve that is established with particles of known size (validated through scanning electron microscopy, SEM). The sizing accuracy of this approach was determined to be ±11 nm^29^. Similarly, we used this phase information to estimate the size of the virus, which acts as a second level of screening in addition to surface chemistry, eliminating the need for any fluorescent labeling. This mobile virus imaging and sensing platform, weighing <500 g, can not only determine the presence or absence of the virus within the sample but can also estimate the viral count of the sample.

## Results and Discussion

**Figure 1:**
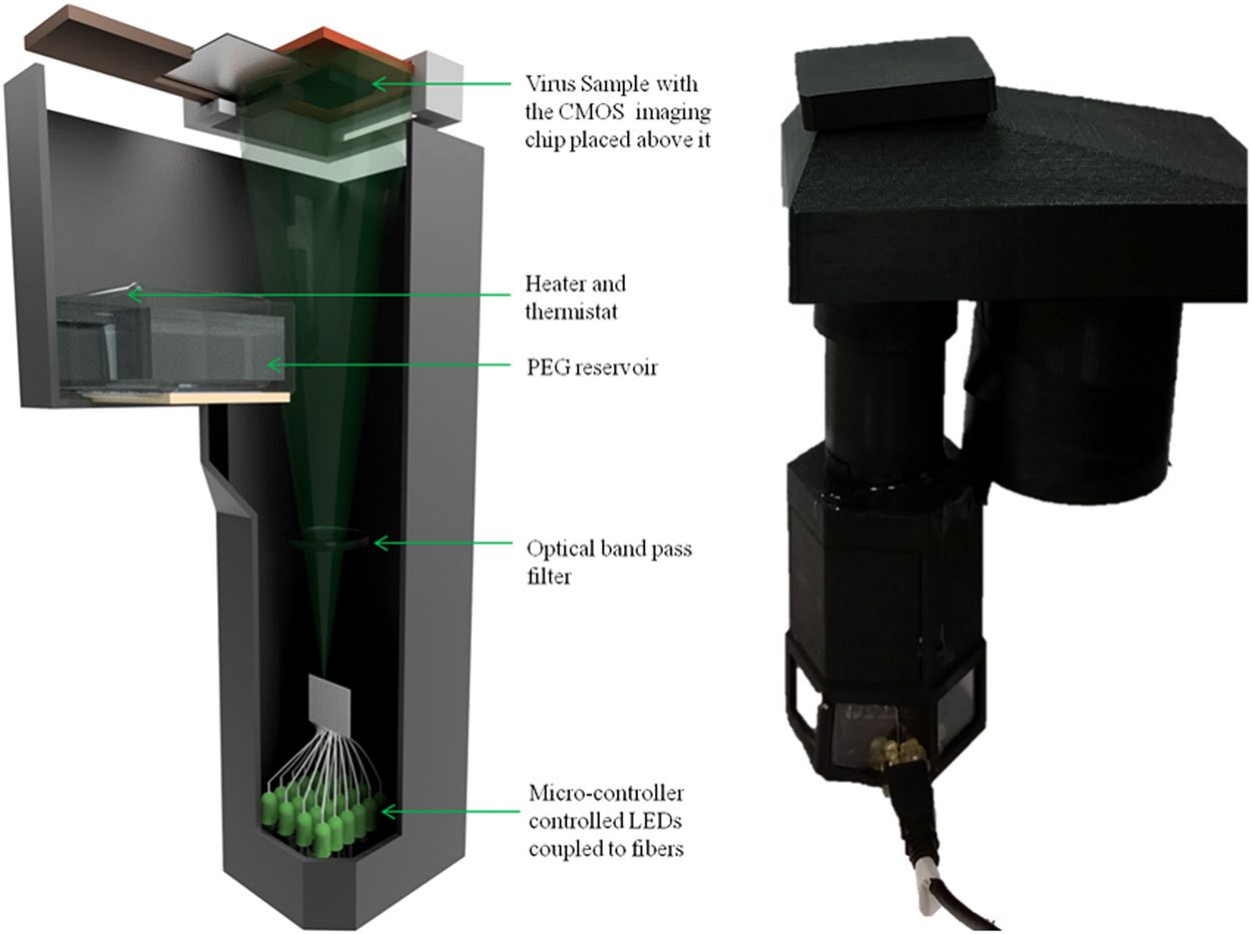
The schematic diagram of the hardware of the portable lensfree microscope is shown on the left. The photograph of the same microscope is shown on the right. This device is about 25 cm in height and weighs less than 500 grams^29^.

**Figure 2:**
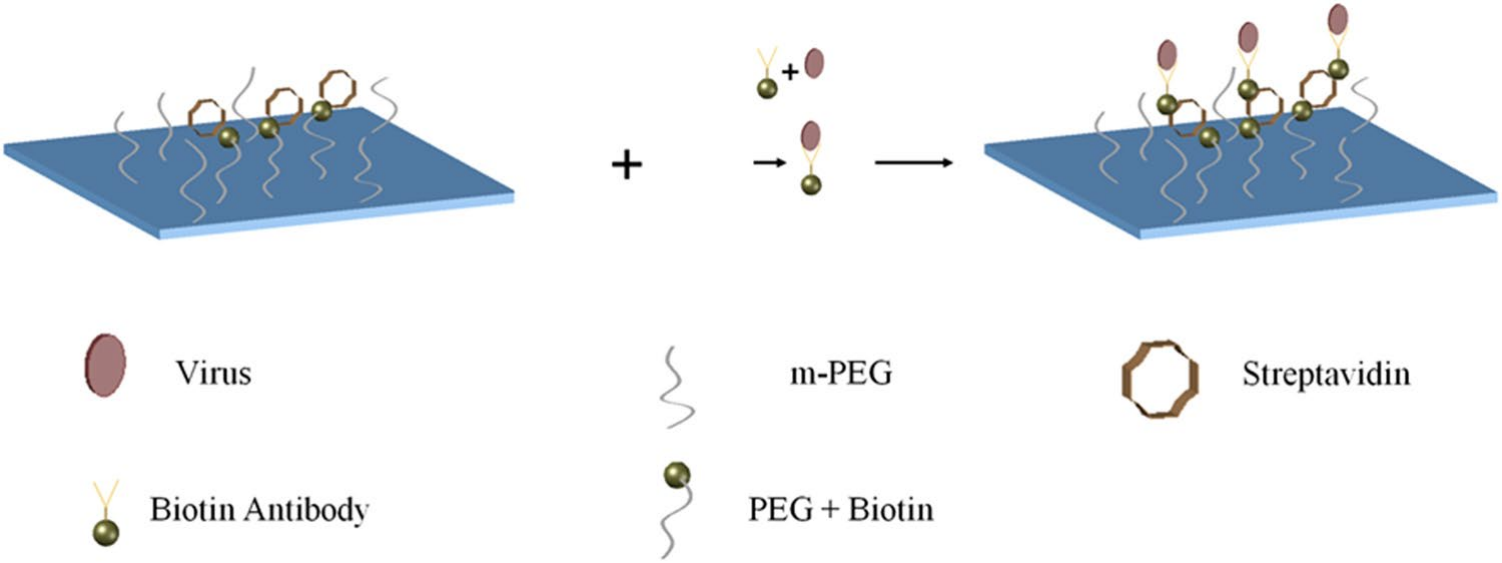
Schematics of the virus capture steps. (**a**) The chip for specific capture of HSV-1 particles is prepared by coating a glass substrate with streptavidin and poly-ethylene-glycol. (**b**) HSV-1 particles in solution are conjugated with biotin tagged antibodies and added onto the substrate. (**c**) HSV-1 particles captured on the substrate are then imaged by our computational holographic microscope for counting their density (counts/mm^2^).

The schematic of the lensfree on-chip holographic microscope is shown in Fig. 1. We used this wide-field holographic microscope for the detection of HSV-1 particles using a two-step process, involving both the immuno-specificity of the viral particles and their physical size properties (~150–200 nm in diameter^17, 34, 35^). This is done by first capturing the target viral particles onto a glass substrate using virus-specific antibodies, as shown in Fig. 2, and as a second step, imaging and sizing of the captured particles to confirm the presence of viruses using our holographic on-chip microscope. The HSV-1 capture is mediated by the biotin-streptavidin conjugation, where the substrate is coated with streptavidin and the viral solution is mixed with biotin tagged HSV antibodies. The nonspecific binding between the virus and the coverslip is reduced by coating the substrate with m-PEG1000.

**Figure 3:**
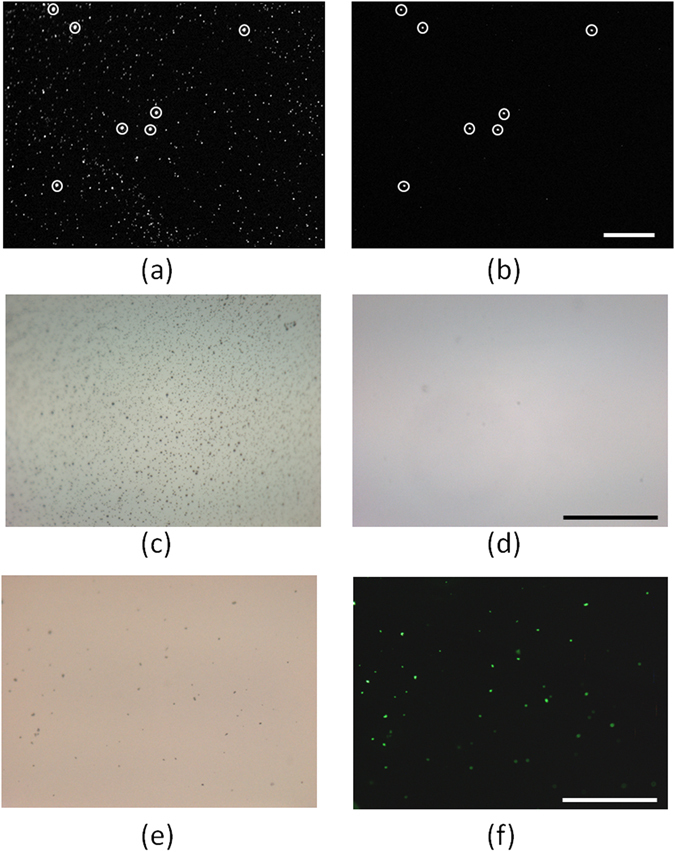
Fluorescence image of 0.25 µm and 1 µm polystyrene beads under (**a**) blue excitation and green emission (green channel), and (**b**) green excitation and red emission (red channel). (**c**) Brightfield image of the virus sample placed on a substrate without the PEG layer. We observe a significant level of non-specific binding. (**d**) Brightfield image of the virus sample without antibody tagging on a substrate with the PEG coating. (**e**) Brightfield image of the virus sample conjugated with biotin tagged antibody and FITC tagged antibody, placed on a substrate with PEG coating. (**f**) The corresponding fluorescence image of the same virus sample (an overlapped RGB image). Scale bar: 100 µm.

To test the capture efficacy of the substrate and the nonspecific binding rate, we performed control experiments using biotin tagged 0.25 µm beads and uncoated 1 µm fluorescent beads, as shown in Fig. 3a,b. The green fluorescence that is observed on the substrate is due to both types of beads, while the red fluorescence is observed only due to non-specific binding of the 1 µm beads. The absorption spectrum of the 1 µm beads is provided in Supplementary Figure S1, showing significant absorption under both 488 nm and 532 nm wavelength excitation. In these results, as desired, we observed a significantly higher ratio of specific binding to nonspecific binding; digital processing of the fluorescent images shows that the number of green fluorescent beads specifically bound to the substrate is about ~ one to two orders of magnitude larger compared to the non-specifically bound red fluorescent beads. A detailed investigation of specific vs. non-specific binding using fluorescent beads is also reported in Supplementary Table 1 as well as Supplementary Figures S2 and S3. Blank images of the substrate with non-fluorescent particles under blue and green illumination are also acquired to demonstrate the specificity of the signal (Supplementary Figure S4). We do not observe any fluorescence signal under either condition.

Next, we tested the specificity of the substrate using HSV-1 particles. This is done in three ways: by using (i) antibody conjugated HSV-1 on unmodified substrates, (ii) HSV-1, without any antibody conjugation, on fully prepared substrates, and (iii) antibody conjugated HSV-1 on fully prepared substrates. When antibody conjugated HSV-1 particles are added to an unmodified glass substrate, without any PEG coverage, we observe a significant amount of nonspecific binding of the virus. The viral particles cover the full surface area of the substrate due to non-specific binding (see Fig. 3c). We next added HSV-1 without any antibody conjugation, onto a fully prepared substrate with PEG and streptavidin coating, and observed no binding of the virus (see Fig. 3d). With the addition of biotin-antibody tagged HSV-1 onto a fully prepared substrate, as expected and desired, we observed a significant amount of virus binding. This was also confirmed by tagging the virus with fluorescently labeled antibodies as an independent validation. The fluorescence image of the labeled viruses and the corresponding bright-field image of the same sample are shown in Fig. 3e,f, respectively. The presence of the viral particles was further confirmed by performing electron microscopy of the samples as shown in Supplementary Figure S5. Previous electron microscopy (SEM and TEM) images have shown similar non-regular structures of the virus, mostly due to partial disruption of the viral envelope during e.g., sample preparation^17, 36, 37^. Note that there are several glycoproteins on the envelop of each HSV particle^3, 34^ that can bind to multiple antibodies, which enable the virus to be labeled with fluorescent molecules and biotin at the same time using multiple antibodies. We conveniently used this approach to cross-validate the efficacy of our surface chemistry technique.

**Figure 4:**
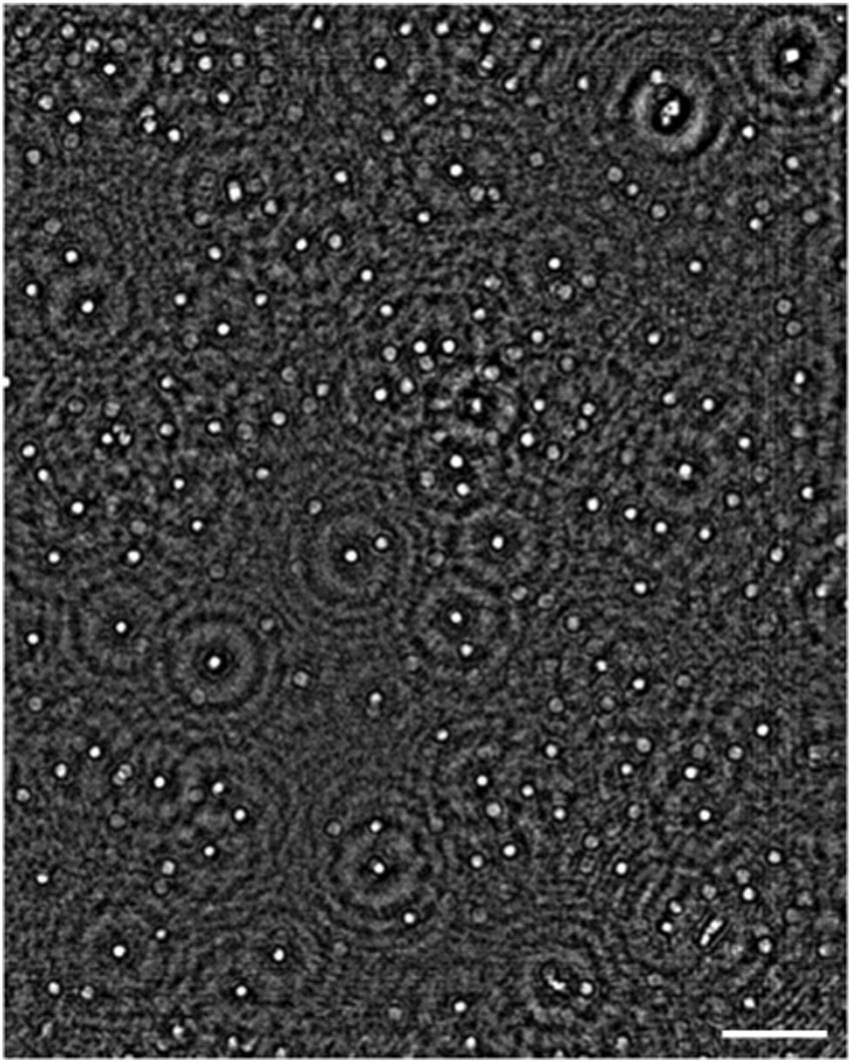
Reconstructed phase image of an HSV-1 sample. Scale bar: 50 µm.

In addition to surface functionalization and immuno-specific capture of the viruses, the specificity of our mobile platform is further improved by digitally incorporating the physical size of the viral particles into the automated decision process. The HSV particle size is approximately 150–200 nm in diameter^17, 34, 35^ and we used this information to digitally reject non-specifically bound particles that are outside of this range as ‘non-viral’, which helps to increase our specificity. Nanoscopic size measurements are typically performed using electron microscopy (which is very accurate but tedious to use and expensive) or dynamic light scattering based approaches (which are limited in accuracy as well as the dynamic range of the particle size and density that can be measured). Our lensfree computational microscopy platform provides a compact and cost-effective method of not only imaging viruses, as shown in Fig. 4, but also sizing them over a very large dynamic range of particle size (~40 nm to mm) and particle density (<1 particle to several thousands of particles per micro-liter)^29^. This range of concentration is clinically relevant for both HSV1 and HSV2, which is on the order of ~10^2^–10^10^ DNA copies per ml depending on the sample and collection method^38^.

**Figure 5:**
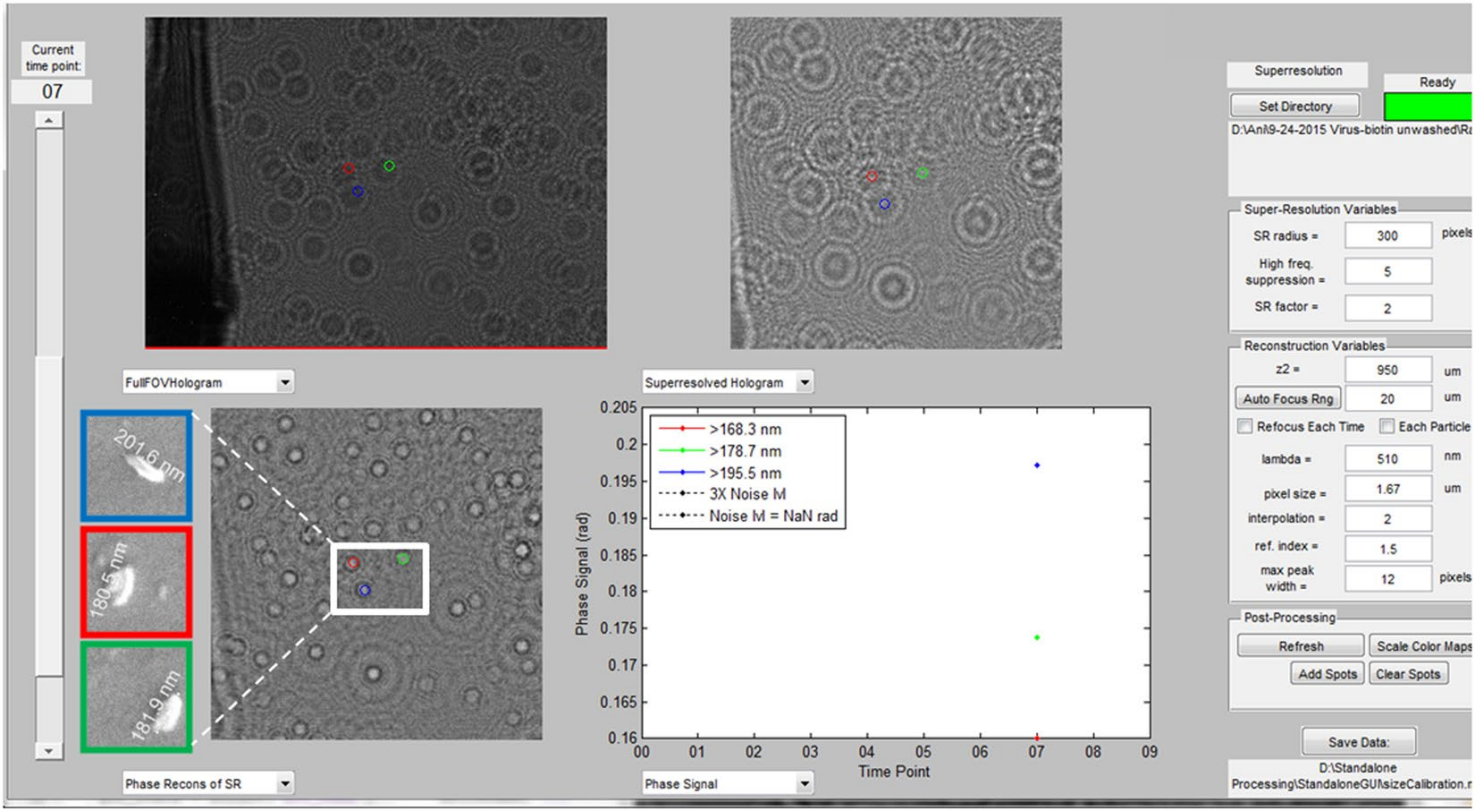
GUI used to reconstruct the holograms of HSV-1 particles and size them. Insets: SEM images of some of the HSV-1 particles, used for comparison purposes. The colors indicate the corresponding viral particle.

**Figure 6:**
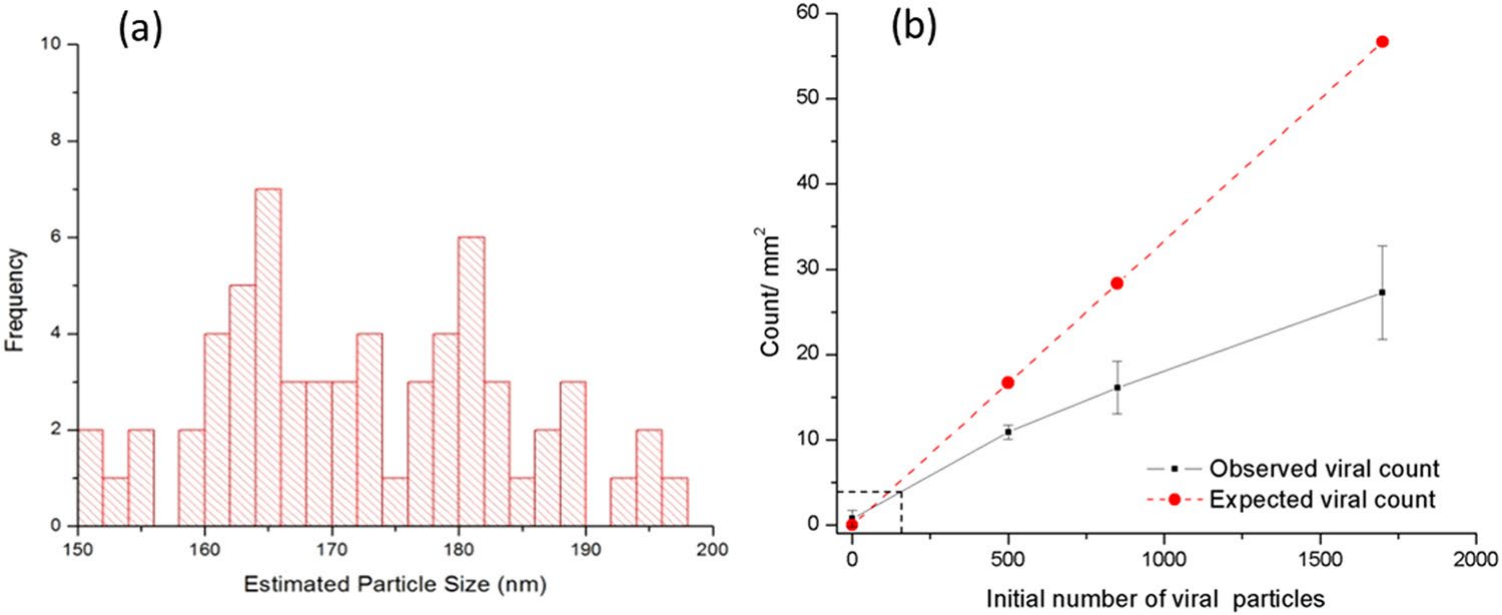
(**a**) Sizing and counting of HSV-1 particles imaged over a FOV of 2.76 mm^2^, with ~1700 viral particles per test, spread over a large FOV of ~30 mm^2^. (**b**) The number of virus particles detected per unit area (mm^2^) of the substrate as a function of the initial number of viral particles per test/assay. The horizontal dashed line refers to μ + 3σ of our control samples, i.e., ~4 counts/mm^2^, which corresponds to ~160 viral particles per test, i.e., the intersection of the vertical dashed line with the initial number of viral particles in (**b**). The std. dev. is obtained from a set of N = 3 measurements.

In our experiments, we performed the measurements using a custom-designed graphic user interface (GUI) by manually selecting a region of interest and then implemented an automated size based counting algorithm on the selected region. In order to illustrate the sizing of the captured virus particles we randomly selected three particles as shown in Fig. 5. Using our computational holographic on-chip microscope, the sizes of these HSV-1 particles are determined to be ~168 nm, ~179 nm and ~196 nm, respectively. The sizing accuracy of this method has been previously established as ±11 nm^29^. We also imaged the same particles using SEM (see Fig. 5 inset) and the results revealed that the measured particle size using SEM is slightly larger than the value measured using our portable microscope, which can be attributed to the additional gold coating on the surface of the virus that is required for SEM imaging. An example of HSV-1 particle size distribution (between 150 nm and 200 nm) that is measured using our approach is also shown in Fig. 6a.

In order to estimate the limit of detection (LOD) of our computational HSV sensor, we measured various samples, each containing a different number of virus particles, with one acting as a control without any viruses. In these experiments, virus particles were suspended within a volume of 50–100 µL per test and we counted HSV-1 particles per unit area (mm^2^) by imaging multiple regions of interest within a given substrate - see Fig. 6b. An estimate of the LOD of our platform is obtained based on the mean (μ = 0.77) and standard deviation (σ = 0.96) of the control samples (i.e., μ + 3σ), which is equivalent to approximately 4 HSV-1 particles per mm^2^ of our FOV (see Fig. 6b). Since our on-chip imaging system has a FOV of ~30 mm^2^, this is equivalent to ~120 counts for the entire area of the CMOS imager chip, assuming 100% recovery rate and that the same sample volume per mm^2^ of chip area is used for each test. These spurious particles and the false positives observed in imaging of the control samples (yielding to ~4 HSV-1 particles per mm^2^) may be due to non-specific nanolens formation on the substrate and/or due to error in our HSV-1 detection, sizing and counting algorithms. Note that if an imager chip with a smaller active area is used, the total volume of the assay will need to be reduced, proportional to the area of the imager chip (assuming that the sample volume per mm^2^ of our FOV remains the same). Therefore, for different on-chip microscope designs that employ other CMOS imagers, the assay volume per test can be accordingly adjusted by simple centrifugation of the sample. Significantly increasing the test sample volume per unit area of the chip would also increase false positives as well as spatial overlap related misses, especially at higher virus concentrations.

In the experimental results reported in Fig. 6b, we observe a relatively low virus recovery rate which can be attributed to several factors. First, the viral conjugation efficiency could pose a limitation. Second, a significant challenge lies in identifying viral clusters by utilizing self-assembled nanolenses. In the presence of such viral clusters or very closely spaced viruses (with a separation of e.g., <300–400 nm), self-assembled nanolens topology starts to get influenced by its neighbors, which might result in sizing errors and missed viral particles; this is also evident in the reduced slope reported in Fig. 6b for higher virus concentrations. Another challenge in our size-based virus counting step is the possibility of disintegration (fragmentation) of some of the viral particles during the test, which may lead to a significant reduction in the size of the viral particles. Viral disintegration may happen during routine handling steps such as centrifugation or pipetting and mixing. Particle recovery rate is also limited by the background noise that randomly appears in some sub-regions of our imaging FOV, created by formation of patches of miniscule droplets, much smaller than our nanolenses. This makes the background hazy and renders some of the areas on the sample chip useless for virus counting. The specific reasons for these patches are unclear, but they have been observed to form due to the increase in surface hydrophobicity because of the virus specific surface chemistry or formation of salt crystals. Over-counting the particles is also possible and can be attributed to non-viral particles in the relevant size range that are present on the substrate. We quantified this by counting the number of particles detected in the sample prepared from blank water solutions with no viruses as illustrated in Fig. 6b, leading to an LOD of ~4 HSV-1 particles per mm^2^ of our FOV. Based on our calibration curve this corresponds to ~160 viruses per test/assay, referring to the initial number of viral particles in the test.

One should note that our experiments were performed using HSV-1 particles from culture. In a clinical setting, the virus is extracted from lesions using swabs and then suspended in a buffer, which can then be used for imaging/sensing using the presented computational microscope. The viral samples can also be concentrated by centrifugation and re-suspended in appropriate amount of buffer to be adjusted per test (e.g., depending on the active area of the imager chip). This computational method of viral particle sensing can be extended to various other types of viruses as well. Counting the number of viral particles in a given sample can not only help determine the severity of the disease but also monitor the efficacy of an anti-viral therapy. One of the important advantages of the presented imaging system is its ability to image over a wide field-of-view (e.g., 30 mm^2^), which could enable multiplexed testing for different viruses in parallel, by e.g., spotting antibodies for a panel of viruses on the same disposable substrate.

## Materials and Methods

### Materials

Streptavidin was obtained from Sigma-Aldrich. Fluorescently labeled beads, biotin tagged Herpes Simplex Virus type 1/2 polyclonal antibody, FITC tagged Herpes Simplex Virus type 1/2 polyclonal antibody, Bovine Serum Albumin and PEG-300 were acquired from Thermo Fischer Scientific. The Biotin-PEG-Silanes, m-PEG-silane were acquired from Creative PEGWorks (NC). The viral culture (NATtrol Herpes Simplex Virus Type 1 Strain: MacIntyre, 50,000 cps) was acquired from ZeptoMetrix Corporation. All the chemicals and materials were used as received.

### Preparation of the substrate

A schematic of the sample preparation method is shown in Fig. 2. The coverslips were thoroughly cleaned by first incubating them overnight in acetone and then rinsing them using ethanol and water. The coverslips are allowed to dry and then plasma treated for 5 minutes to further clean the surface and also make them hydrophilic. The substrates were prepared by first adding a mixture of mPEG (38 mg) and biotin-PEG (2 mg), in a solution of 95% ethanol (1875 µl) and acetic acid (125 µl). After an hour of incubation the coverslips were washed with ethanol and water to remove any unreacted PEG. We repeat this procedure twice to ensure full surface coverage with PEG, to reduce any non-specific binding. After completion of PEGylation, we add streptavidin (~100 µg/ml) and incubate it for an hour. Any excess unbound streptavidin is washed off using water. The streptavidin coated coverslips can be used immediately or stored in the freezer for future use and testing. The virus solution in buffer was mixed with biotin tagged antibodies and gently mixed in a shaker for ~1–2 h. This is also in agreement with previously reported experimental protocols on labeling of HSV-1 particles, performed by antibody incubation for ~1 h^39^. The antibodies were used at a dilution of 1:20 as specified by the company. The same step can be similarly performed in serum. The solution was then centrifuged to remove any unreacted antibody and re-suspended in water. The antibody conjugated virus was then added to the streptavidin coated coverslip as a droplet, and incubated for more than an hour for the biotin to bind with streptavidin. The sample is then washed thoroughly with water following this incubation step. The droplet is approximately the size of the sensor area. The volume of the viral solution per test was varied from ~50 µl to a maximum of 100 µl, depending on the concentration of the initial solution. Varying the test volume in this small range does not significantly affect the number of false positives in the size range of HSV (~150–200 nm) (see Supplementary Figure S6). The range of volume used in our experiments is similar to the sample volume used in other techniques such as ELISA, PCR etc.^9, 18^. The desired viral concentration is achieved by diluting the virus stock solution. To estimate the error in this dilution process, we performed the same experimental protocol with serially diluted fluorescent beads and measured the optical absorbance and fluorescence using a plate reader. We observed about ~2% variation in the signal intensity between the wells, which should also apply to our viral dilution process as the same protocols were used.

### Holographic Microscope

The schematic of our lensfree holographic microscope is shown in Fig. 1. The microscope was designed using custom-made 3D printed parts. This super resolution holographic imaging system consists of 20 LEDs coupled to optical fibers (with 0.1 mm core diameter, Thorlabs, AFS-105/125Y) individually controlled using a micro-controller (Atmel, ATmega8515), an optical band pass filter (at 510 nm, having a bandwidth of 10 nm, Semrock Inc., FF02-510/10–25), a sample coverslip holder and a 10 mega-pixel CMOS image sensor chip with USB readout (Imaging Development Systems, UI-1492LE-M). Of the 20 LEDs, we used eighteen green LEDs (~527 nm), one red LED (~625 nm) and one blue LED (470 nm)^23^. The sample was illuminated from the bottom using LEDs that are sequentially turned on and off, and the transmission holograms of the virus sample are recorded using the CMOS camera, which is placed at a distance of <1 mm from the sample plane. The sub-pixel shifted holograms are then utilized to generate a pixel super-resolved hologram, which is then reconstructed to obtain the phase and amplitude images of the sample^23, 25^. The entire imaging system was controlled using a custom-developed LabVIEW program.

Self-assembled liquid nanolenses were deposited on the sample from a reservoir containing PEG-300, attached to the imaging system. The PEG-300 solution is heated to the desired temperature using a resistive heater (Omega Engineering, KHLV-101/10), placed inside the reservoir. The temperature is set and monitored using a computer-controlled feedback temperature controller, with a thermistor (TE Technology, Inc. TC-48-20) immersed in the PEG solution. The temperature control unit is interfaced to the main PC through a LabVIEW program as well.

The experiments are performed by first acquiring a set of images before the PEG condensation, which serves as a baseline to the evaporation process. The temperature of the PEG is then increased to ~95 °C, which results in evaporation and subsequent PEG deposition/condensation on the sample and formation of the nanolenses. The lensfree holographic images are continuously acquired during this process to monitor the evolution of the nanolenses and to determine the optimal time point for virus detection. Image reconstruction and analysis using our GUI takes only a few minutes. Following the completion of the imaging process, at the sample we observe a small temperature change of about 4 °C, from 27 °C to 31 °C (Supplementary Figure S7).

In our experiments, we observed that sub-regions of the substrate randomly exhibit patches with high density of PEG droplets also discussed as part of our Results section. These randomly formed patches on the substrate prevent proper identification and counting of the target nanoparticles or viruses. For the purpose of this study we avoided using these regions in our analysis. This is done by identifying the individual holograms formed by the viruses and nanoparticles (after the nanolens formation), while eliminating other regions. First, we used an edge detection algorithm and outlined the edges of the holograms. Then we performed Hough transformation to spot out the circularly shaped holograms. These selected areas are used for virus detection and counting (per unit area) and the rest of the FOV is discarded.

The automated viral particle counting is performed using a segmentation algorithm^29^, where the holographic image reconstruction and all the digital processing steps are performed using a custom developed MATLAB program running on a PC (Dell Optiplex 9010, 32 GB RAM memory, Intel core i7 processor @3.4 GHz). After inputting the correct holographic reconstruction parameters, including the wavelength and the effective pixel size, the algorithm first reconstructs pixel super-resolved images of the sample at different sample-to-sensor distances (z_2_). Using this stack of z_2_ images, some spurious particle-like noise features are eliminated by incorporating a focusing criterion for particle identification. Stated differently, real particles show a gradual change in their phase signal as a function of z_2_, whereas particle-like noise terms do not exhibit a similar focusing behavior. We used this physical focusing criterion to remove spurious particle-like features, enabling proper identification and counting of the specifically-captured viral particles. In this digital counting process, the larger particles, which have higher peak phase values in our reconstructed phase images, are counted first. Then, they are digitally removed along with their twin images, which often mask the smaller particles located in close proximity to larger particles^29^. Such larger particles are digitally removed by replacing their amplitude and phase information with the average of the background in the twin-image plane. The above described steps are repeated multiple times till all the particles are automatically counted.

### Fluorescence Microscopy

A bright-field and fluorescence microscope (Olympus, BX51) was used for comparison purposes. These comparison images were acquired with a scientific charge-coupled device (CCD) camera (Retiga 2000R, QIMAGING) by optimizing the gain and exposure time of the camera for each modality. Fluorescence imaging of the viral particles was performed by first tagging the virus with FITC conjugated antibodies. These HSV-1 particles were imaged using a 40× objective lens and the sample was irradiated with 488 nm excitation light and the fluorescence light was observed at ~532 nm.

The specific binding studies were performed using two different fluorescent beads. 0.25 µm green fluorescent beads with biotin coating and 1 micron red fluorescent beads were mixed in equal amounts and deposited on the substrate. After an hour of incubation the beads were thoroughly washed using water and imaged under the fluorescence microscope. The green fluorescent beads were imaged using the 488 nm excitation filter and 532 nm emission filter, whereas the red beads were imaged using the 532 nm excitation filter and the 632 nm emission filter.

### Scanning Electron Microscopy

To provide image comparisons, some of the virus samples under test were first coated with gold using a sputter coater (Denton Desk V) with a thickness of around 10 nm. The samples were then imaged using an SEM (Nova 600 SEM/FIB System). The following steps were performed to register the images, which is used for comparison purposes. First, easily identifiable marks were placed on the sample slides, before imaging with the lensfree platform. These marks are clearly visible in the reconstructed images, and during SEM imaging (even after metal coating), serve as the main anchors for image registration. Prior to SEM imaging, secondary anchor points (larger particles that are easier to register) are identified near the target particles. The horizontal and vertical distances of these targets with respect to the secondary anchors are calculated from the reconstructed image to form a roadmap. During the SEM imaging, the sample is first aligned such that the angular orientation of the slide matches the reconstructed image. Once the secondary anchors are identified, each target particle is visited by shifting the sample holder by appropriate distances. This step-by-step particle searching strategy enables us to register our images accurately, which is used for comparison.

## Electronic supplementary material

Supplementary Information
