# Supplementary material for: Computational sensing of herpes simplex virus using a cost-effective on-chip microscope
Source: Sci Rep. 2017 Jul 7;7:4856. doi: 10.1038/s41598-017-05124-3 (PMC5501859; doi:10.1038/s41598-017-05124-3)
Supplement: Supplementary file 1 — Supplementary Information [file 41598_2017_5124_MOESM1_ESM.pdf]

Supplementary Information

Computational sensing of herpes simplex virus using a cost-effective on-chip microscope

Aniruddha Ray[1,2,3], Mustafa Ugur Daloglu[1,2,3], Joslynn Ho[2], Avee Torres[4], Euan Mcleod[5] and Aydogan Ozcan[1,2,3,6]**

[1]Electrical Engineering Department, University of California, Los Angeles, CA, 90095, USA

[2]Bioengineering Department, University of California, Los Angeles, CA, 90095, USA

[3]California NanoSystems Institute (CNSI), University of California, Los Angeles, CA, 90095, USA

[4]Department of Physics, University of California, Los Angeles, CA, 90095, USA.

[5]College of Optical Sciences, University of Arizona, Tucson, AZ, 85721, USA.

[6]Department of Surgery, David Geffen School of Medicine, University of California, Los Angeles, CA, 90095, USA

*Corresponding Authors: rayani@ucla.edu ; ozcan@ucla.edu

Analysis of non-specific binding: Non-specific binding was quantified using two types of fluorescent beads, biotin coated (0.25 µm) and uncoated (1 µm and 0.25 µm). The beads, at different concentrations, were incubated on the prepared substrates and then thoroughly washed to remove the unattached beads. We observed approximately ~5 to 12 times more specific binding compared to nonspecific binding as shown in Supplementary Figure S2 and Supplementary Table 1. There was no statistically significant difference between the binding of 0.25 µm and 1.0 µm uncoated beads onto the substrate. We then used a mixture of biotin coated green fluorescent beads and uncoated orange beads (1.0 µm) and incubated them on the substrates. The fluorescence images of the beads are shown in Supplementary Figure S3. We observe a significant increase in the binding ratio (e.g., ~20-80 times higher specific binding vs. non-specific binding). This increase in ratio may be attributed to the decrease in surface area or binding sites, such as free biotin or streptavidin, available to the uncoated beads in the presence of biotin coated beads.

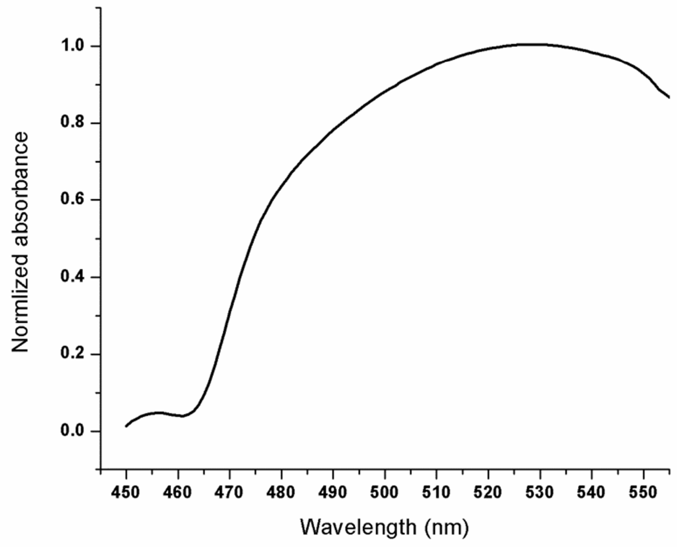

Supplementary Figure S1: The absorption spectrum of 1 µm beads used in our experiments.

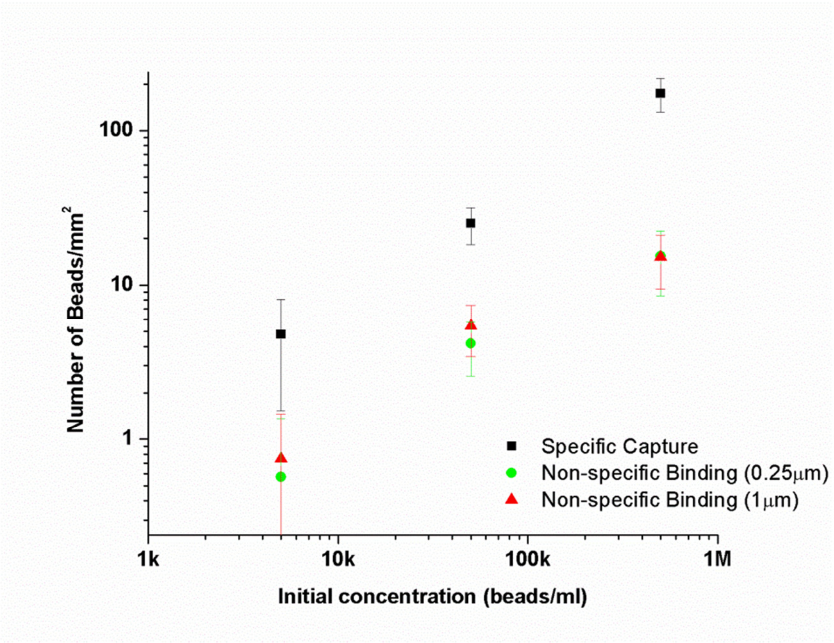

Supplementary Figure S2: The plot of the number of fluorescent beads detected per unit area (mm^2) due to specific binding and non-specific binding (beads used: 1 μm and 0.25 μm, the latter size was used for both specific and non-specific binding experiments, corresponding to black and green data points, respectively, in the figure). The concentration of the beads is similar to the concentration of viral particles, i.e., following a clinically relevant range. These experiments were performed by depositing only one type of bead on each prepared substrate. For each experiment, a sample volume of 50 μL was used and spread over the entire imaging field12 of12view, ~30 mm^2. The std. dev. is obtained from a set of N=5 measurements.

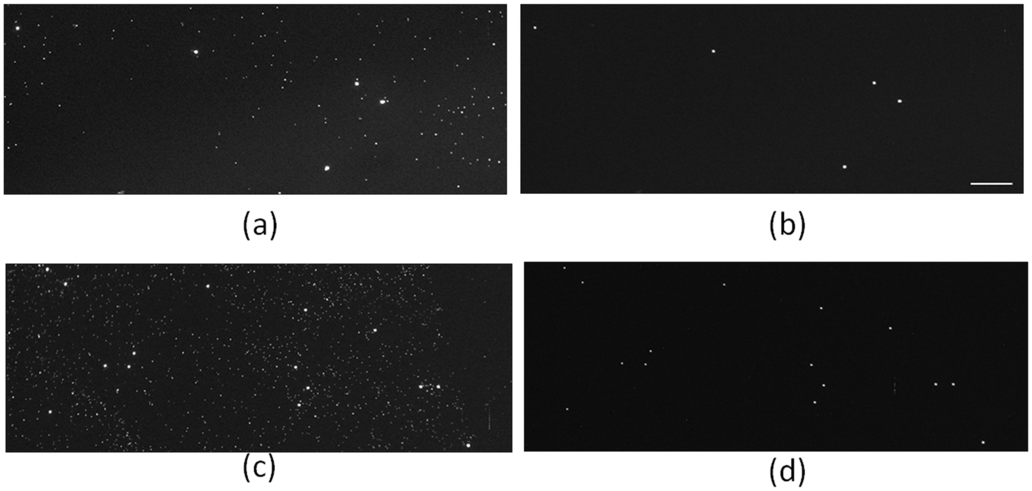

Supplementary Figure S3: Fluorescence images of beads (0.25 μm and 1μm) bound to the substrate. (a,c) Blue excitation and green emission filter (green channel). (b,d) Green excitation and red emission filter (red channel). These experiments were performed by mixing two types of beads (biotin coated 0.25 μm beads and uncoated 1μm beads) at equal concentrations and depositing them on the substrate. Figures (a & b) show the captured beads for a concentration of ~10^6 beads/ml. We observe a slightly higher ratio (~20) of specific binding to non-specific binding compared to the previous experiment with ~ 5×10^5 beads/ml, where the beads were deposited separately (see Supplementary Figure S2 and Supplementary Table 1). When we increase the bead concentration to ~10^8 beads/ml, the ratio of specific to non-specific binding increases to ~80 (c & d). Scale: 100 μm.

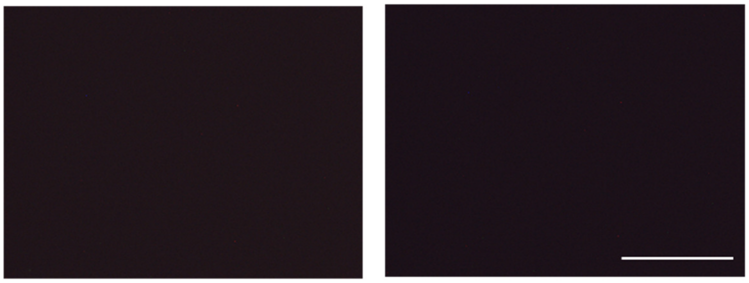

(a) (b)

Supplementary Figure S4: Images of non-fluorescent beads under (a) blue excitation and green emission filter and (b) green excitation and red emission filter. We do not observe any fluorescence signal in either of the channels. (Scale: 100 μm)

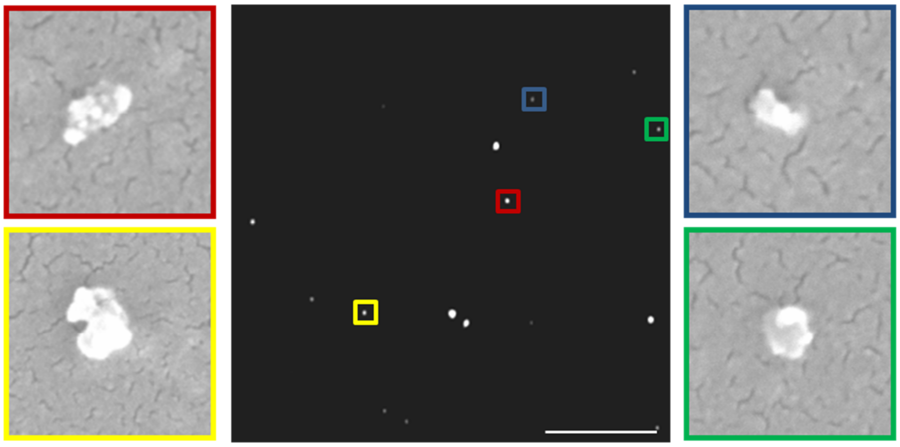

Supplementary Figure S5: Fluorescence image of FITC antibody tagged HSV (green channel) and the corresponding SEM images of selected particles (insets) (Scale bar: 100μm). The colors indicate the corresponding viral particles. The insets are 500nm x 500nm in size.

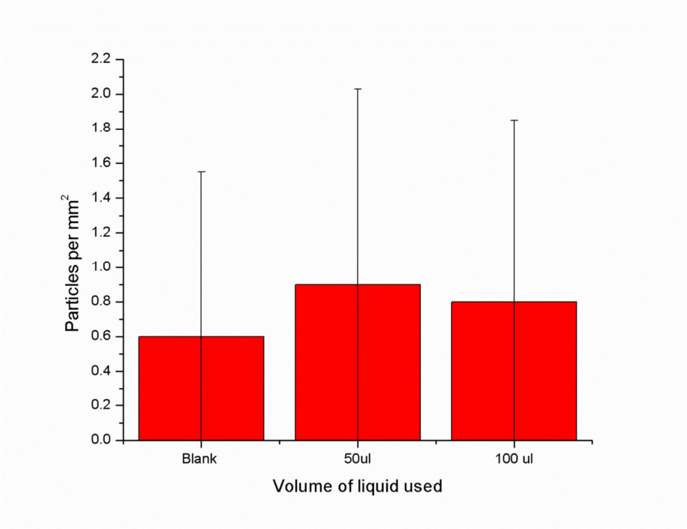

Supplementary Figure S6: Number of spurious particles detected per mm^2 of the substrate for control samples (i.e., without any viruses). Blank refers to bare substrates with no sample or solution. The other two control experiments refer to 50 and 100 µl of water solution per test.

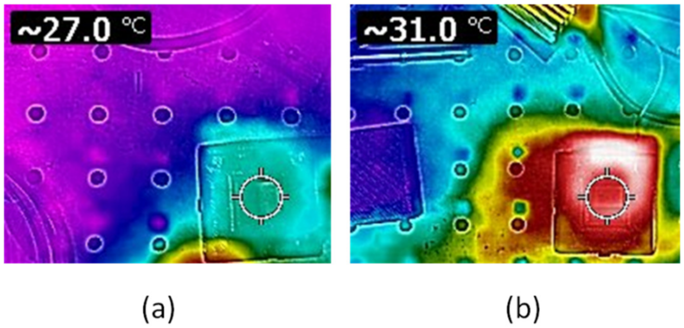

Supplementary Figure S7: The temperature of the sample (a) before and (b) immediately after performing holographic imaging. We observe a 4 °C change in the sample temperature. The temperature readings were performed on the glass substrate (in the region indicated by the white circle) which was placed on a sample holder during imaging. The images are acquired using a thermal camera (FLIR C2, FLIR systems AB, Sweden).

	5k beads/ml	50k beads/ml	500k beads/ml
Specific binding	4.8 ± 3.3	25 ± 6.8	175 ± 44
Non-specific binding (0.25μm)	0.6 ± 0.8	4.1 ± 1.6	15.4 ± 6.9
Non-specific binding (1.0 μm)	0.7 ± 0.7	5.4 ± 2.0	15.2 ± 5.7

Supplementary Table 1: The number of fluorescent beads detected per mm^2 due to specific binding and non-specific binding as a function of the initial bead concentration. These values are also plotted in Supplementary Figure S2. For each experiment a sample volume of 50 μL was used and spread over the imaging field-of-view, ~30 mm^2. The std. dev. is obtained from a set of N=5 measurements.
